# Predominance of multi-drug resistant bacterial pathogens causing surgical site infections in Muhimbili national hospital, Tanzania

**DOI:** 10.1186/1756-0500-7-500

**Published:** 2014-08-07

**Authors:** Joel Manyahi, Mecky I Matee, Mtebe Majigo, Sabrina Moyo, Stephen E Mshana, Eligius F Lyamuya

**Affiliations:** 1 Department of Microbiology/Immunology, Muhimbili University of Health and Allied Sciences, P. O. Box 65001, Dar es Salaam, Tanzania; 2 Department of Microbiology/Immunology, Catholic University of Health and Allied Sciences, P. O. Box 1464, Mwanza, Tanzania

**Keywords:** Multidrug resistance, Surgical site infections, Tanzania

## Abstract

**Background:**

Surgical site infections (SSIs) remain a common and widespread problem contributing to a significant morbidity and mortality, attributed partly by the increase in antimicrobial resistance among the etiological agents. This study was done to determine the spectrum of bacterial isolates and their susceptibility patterns causing SSIs at Muhimbili National Hospital, Tanzania.

**Methods:**

This descriptive cross sectional study was conducted between September, 2011 and February, 2012. Pus swabs or pus were cultured on blood agar (Oxoid, UK) and MacConkey agar (Oxoid, UK) and incubated aerobically at 37°C for 18–24 hours. Bacterial identification was done using API 20E and VITEK and antimicrobial susceptibility was determined by Kirby Bauer disc diffusion.

**Results:**

Of the 100 patients, from whom wound swabs were collected, 90 (90%) had positive aerobic bacterial growth. A total of 147 pathogenic bacteria were isolated, including 114 (77.5%) gram negative and 33(22.5%) gram positive organisms. The most prevalent bacterial species were *Pseudomonas aeruginosa* (16.3%), followed by *Staphylococcus aureus* (12.2%) and *Klebsiella pneumoniae* (10.8%). Of the 18 *S. aureus* , 8 (44%) were methicillin resistant *Staphylococcus aureus* (MRSA) and three of them (17%) were carrying both MRSA and induced clindamycin resistance (ICR). Extended spectrum beta-lactamase (ESBL) producing Enterobacteriaceae were observed in 23 (79.3%) of the 29 isolates tested. Majority of *Escherichia coli* 12 (92.3%) and *K. pneumoniae* 11 (69%) isolates were ESBL producers. About 63% (93/147) were multiple-drug resistance (MDR) isolates, and the overall MDR among Gram positive and Gram negative bacteria was 60.6% (20/33) and 61.4%, (73/114), respectively. The prevalence of MDR for *E. coli*, *A. baumannii* and *P. stuartii* was 100% each. Majority (97%) of the Gram negative bacteria were resistant to more than four categories (classes) of antibiotics.

**Conclusion:**

A high proportion (63%) of the isolates causing SSIs in this tertiary hospital were MDR, of which (90%) were resistant to more than four classes of antibiotics. In the light of these findings, an urgent and significant change in antibiotic prescription policy is required at this National hospital.

## Background

Surgical site infections (SSI) remain a common and widespread problem that contributes to significant morbidity and mortality, prolongs hospital stay and consequently increasing health care cost [1]. Globally, SSI is reported to be the third most common nosocomial infection preceded only by urinary tract infections and pneumonias [1,2]. The outcome of SSI is partly attributed to increase in antimicrobial resistant bacterial pathogens [3], which make the choice of empirical therapy more difficult. Globally, the incidence of SSIs may range from 1.5 – 20% depending on the diversity and complexity of procedures performed, age of patients, pre-operative hospitalization, length of surgery as well as geographical locations [4,5].

SSI remains the major cause of nosocomial infections in Tanzania that imposes substantial burdens on healthcare resources [6]. Recent studies reported increase of SSI ranging from 19.4% to 36.5% [7-10], being higher at Muhimbili National Hospital (MNH) compared to other referral hospitals [10]. In these studies *Staphylococcus aureus* was reported to be a predominant pathogen followed by *Escherichia coli*, and *Klebsiella* species with varying antimicrobial susceptibility patterns. A study at Bugando Medical Centre (BMC) reported resistance to ciprofloxacin of 86%, 80% and 54% for *E. coli*, *Klebsiella pneumoniae* and *S. aureus* respectively. Of the *E. coli* and *K. pneumoniae* involved in SSI at Bugando Medical Centre 65% and 80% were ESBL producers, respectively [7]. Rural settings have not been spared of the antibacterial drug resistance problem as shown by a study done (2004) at a remote district hospital in Tanzania which reported more than 95% of *S. aureus* isolates being resistant to penicillin with 1(0.8%) being resistant to methicillin [8]. However, recent data from the same geographical location has revealed significant increase of methicillin resistant *S. aureus* (MRSA) (18.8%) causing SSI [7].

Previous studies conducted in Tanzania have shown persistently high levels of antimicrobial resistance in bacteria isolated from urine and bloodstream infections [11,12]. However, there is rather limited data regarding the magnitude and pattern of antimicrobial resistance among pathogens causing SSI. We undertook this study at MNH, which is the national referral hospital, and where antimicrobial use is probably the highest to determine antimicrobial resistance pattern, including multidrug resistance, among bacteria causing SSI.

## Methods

### Study design and study setting

This was a descriptive cross sectional study performed between September, 2011 and February, 2012 at the Muhimbili National hospital (MNH). Located in Dar es Salaam, MNH is a 1400-bed facility and the largest hospital in Tanzania, which attends 1000 to 1200 outpatients a week and serves approximately four million people living in the Dar es Salaam region. MNH is the main referral and teaching hospital.

### Patient recruitment

All patients with clinical evidence of SSI as defined by the Centers for disease control and prevention (CDC) [13] were eligible for the study. Recruitment was done after obtaining an informed consent. A total of 100 patients were enrolled consecutively from general surgery, obstetrics and gynecology, and orthopedic wards.

### Data collection and laboratory procedures

Structured standard questionnaires were used to obtain social-demographic and clinical data from patients and the patient’s case notes. Thereafter two pus swabs or pus were collected aseptically from the depth of the wound and transported immediately to the laboratory in Amies transport media (Oxoid, UK) for processing.

Gram’s stain was performed from the first swab while the second pus swab was inoculated into blood agar (Oxoid, UK) and MacConkey agar (Oxoid, UK); and incubated aerobically at 37°C for 18–24 hours. Identification was based on microscopic characteristics, colonial characteristics, and Biochemical tests as described by Murray et al. [14], including VITEX (BioMerieux, France) and API 20E (BioMerieux, France). Gram negative organisms were identified by oxidase, Triple sugar Iron (TSI), sulphur indole and motility (SIM), urease, citrate test, VP and Methyl red test. Whereas Gram positive organisms were catalase reaction, coagulase test, DNase test and bile esculin test.

### Antimicrobial susceptibility testing

This was performed by Kirby Bauer disc diffusion method as described by Clinical and Laboratory Standards Institute [CLSI] [15]. Briefly test and control organisms (listed below) were suspended in normal saline to McFarland 0.5 standard and inoculated on Mueller-Hinton agar [Oxoid, UK]. Appropriate discs were placed onto the media then incubated at 37°C for 24 hrs. For Gram positive organisms discs included penicillin (10 units), ampicillin (10 μg), amoxicillin/clavulanate (20/10 μg), ceftriaxone (30 μg), gentamicin (10 μg), erythromycin (15 μg), tetracycline (30 μg), ciprofloxacin (5 μg), clindamycin (2 μg), trimethoprim/sulphamethaxazole (1.25/23.75 μg) and chloramphenicol (30 μg). The Gram negative organisms were tested against ampicillin (10 μg), amoxicillin/clavulanate (20/10 μg), ceftriaxone (30 μg), ceftazidime (30 μg), cefotaxime (30 μg) gentamicin (10 μg), tetracycline (30 μg), ciprofloxacin (5 μg), trimethoprim/sulphamethaxazole (1.25/23.75 μg), chloramphenicol (30 μg) and meropenem (10 μg). For *P. aeruginosa* discs that were used included ceftriaxone (30 μg), ceftazidime (30 μg), gentamicin (10 μg), tetracycline (30 μg), ciprofloxacin (5 μg) and meropenem (10 μg).

MRSA was determined by disc diffusion test using cefoxitin (30 μg) disc on Mueller –Hinton Agar, incubated and maintained at 33-35°C for 24 hours, zone of inhibition ≤ 21 mm was considered a positive result for MRSA strain [15]. Induced clindamycin resistance among *S. aureus* was detected by disc diffusion method on Mueller-Hinton Agar, where 15 μg of erythromycin disc and 2 μg of clindamycin disc were spaced 15 mm apart, incubated at 35 ± 2°C for 16–18 hours. Flattening of the zone of inhibition adjacent to erythromycin (D-zone) was interpreted as ICR [15]. ESBLs production was screened by disc diffusion on Mueller-Hinton Agar with ceftazidime (30 μg) or cefotaxime (30 μg) and confirmed by double disc approximation method [15,16]. Quality of media, antibiotic disc as well as performance of a person carrying the tests was controlled by reference strains: *Escherichia coli* ATCC 25922, *K. pneumoniae* ATCC 700603 for ESBL, *S.aureus* ATCC 25923, and *S.aureus* ATCC 29213 for MRSA.

Multidrug resistant (MDR) was defined as acquired non-susceptibility to at least one agent in three or more antimicrobial categories [17]. Antimicrobial categories tested were Penicillin class (ampicllin, penicillin); Cephalosporin class (ceftazidime, cefotaxime, ceftriaxone); Aminoglycosides class (gentamicin); Tetracycline class (Tetracycline); fluorquinolones class (ciprofloxacin); folate pathway inhibitors class (co-trimoxazole); phenicols class (chloramphenicol); macrolides class (erythromycin) and lincosamides class (clindamycin).

### Data analysis

Data were entered and analyzed using Epidata Entry version 3.1 and STATA version 11.2, and expressed in a descriptive analysis. Categorical variables were compared using Chi-square test or fisher exact test, p values of < 0.05 was considered significant.

### Ethical approval

Ethical approval was obtained from the Senate Research and Publications Committee of Muhimbili University of Health and Allied Sciences, Dar es Salaam and a written informed consent was obtained from each patient/caretaker.

## Results

### Demographical – clinical characteristics

We enrolled a total of 100 patients with clinical SSI, aged between 18 and 80 years. Most of the patients (53%) were males and nearly half (46%) were from orthopedic and trauma wards. Regarding surgical procedures, 27% of the patients had surgical debridement and external fixation, 25% had laparotomy while 15% had caesarian section (see Additional file 1). Almost all patients (95%) had documentation of antimicrobial exposure within a month, with ceftriaxone, cloxacillin and amoxicillin-clavulanic acid the most frequently used, being prescribed to 80 patients each, followed by gentamicin (19), ciprofloxacin (17) and metronidazole (15). Notably, three-fourth had a history of previous admission within 6 months.

### Etiology of SSI

**Figure 1:**
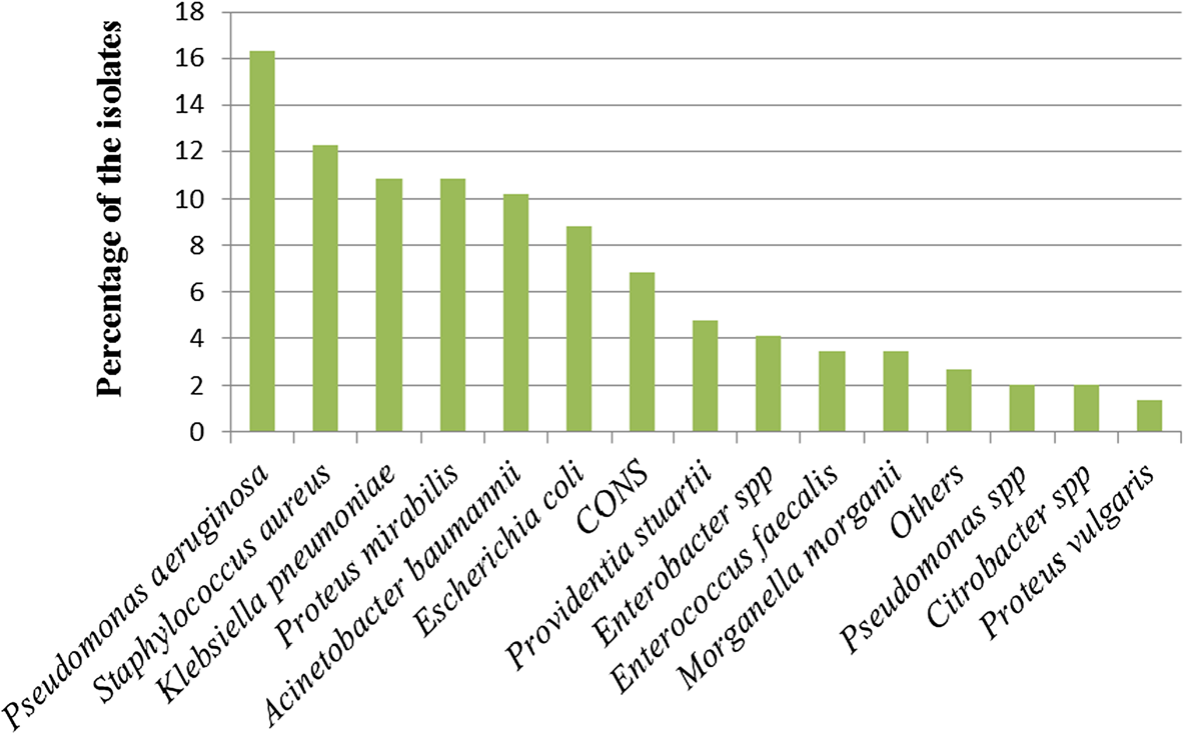
Isolation frequency of pathogenic bacterial isolates from post-operative wound infections (N = 147).

Of the 100 wound swabs collected, 90% had bacterial growth. More than half (52.2%, 47/90) had pure bacterial growth (mono isolate). Gram negative organisms were more prevalent than gram positive bacteria accounting for 77.5% (114/147) of all isolates. The most predominant Gram negative organism was *P. aeruginosa* comprising 16.3% (24/147) of all bacterial isolates. *K. pneumoniae* 10.8% (16/147) and *Proteus mirabilis* 10.8% (16/147) were the next two common Gram negative organisms [Figure 1]. Most (92.3%) of the 13 *Escherichia coli* and 11/16 (69%) of *K. pneumoniae* isolates were ESBLs producing strains. Of the Gram positive isolates, *S.aureus* was the leading cause of SSIs accounting 12.2% (18/147) of all isolates, followed by coagulase negative Staphylococci (6.8%) and *Enterococcus faecalis* (3.4%). Of the 18 *S. aureus* isolates, 44.4% (8/18) were MRSA strains and three of them had induced clindamycin resistance (ICR).

### Antimicrobial resistance pattern

Most of *S. aureus* isolates were highly resistant (83%) to penicillins group of antibiotic; some had low to moderate resistance (17% to 33%) to erythromycin, gentamicin and ciprofloxacin. All Enterobacteriaceae displayed high magnitude of resistance (69% to 100%) to multiple antibiotics tested but all were sensitive to meropenem; and 20-56% were resistant to ciprofloxacin. Surprisingly, 92% of 24 *P. aeruginosa* isolates were sensitive to both gentamicin and ciprofloxacin. Majority of *A. baumannii* isolates were highly resistant (73% to 100%) to most antimicrobial agents; however 60% of them appeared to be moderately sensitive to imipenem [Table 1]. The magnitude of resistance to gentamicin, ciprofloxacin, co-trimoxazole and tetracycline was extremely higher in organisms carrying MRSA (44.4%) and ESBL (79.3%) combined than in non-carrying isolates [p < 0.001]. Of all isolates 63% (93/147) were MDR strains, the overall prevalence of MDR strains was 60.6% among Gram positive and 61.4% among the Gram negatives. MDR status was displayed in all *Escherichiacoli*, *A. baumannii* and *P. stuartii*, and 87.5% of *Klebsiella spp*. Surprisingly, only one isolate of the 24 *P. aeruginosa* displayed MDR [Table 2].

**Table 1:** Antimicrobial resistance pattern of bacteria isolated from post-operative SSIs

**Bacteria isolates (N)**	**Antimicrobial agents resisted (%)**													
	**P**	**AP**	**AMC**	**CRO**	**CAZ**	**CTX**	**C**	**TS**	**TE**	**E**	**CD**	**CN**	**CP**	**MEM**
***E. coli *** **(n = 13)**	NA	100	92	92	92	92	42	85	85	NA	NA	92	58	0
***K. pneumonia *** **(n = 16)**	NA	100	94	81	88	88	54	94	56	NA	NA	67	56	0
***P. mirabilis (*** **n = 16)**	NA	73	87	69	67	100	69	69	94	NA	NA	63	56	0
***A. baumannii *** **(n = 15)**	NA	100	100	100	86	100	100	77	73	NA	NA	86	47	40
***P. aeruginosa *** **(n = 24)**	NA	NA	NA	88	21	NA	NA	NA	NA	NA	NA	8	8	0
**Other G – (n = 30)**	NA	90	83	83	57	76	73	77	65	NA	NA	38	47	0
***S. aureus *** **(n = 18)**	83	92	73	47	NA	NA	20	35	39	17	6	33	29	NA
**Other G + (n = 15)**	67	40	67	50	NA	NA	50	42	47	79	25	62	65	NA

G-: Gram negative bacteria [*P.stuartii, M.morgannii, P.vulgaris, Enterobacter spp, Citrobacter spp*), G+: Gram positive cocci [CoNS, *E.faecalis*]. P = Penicillin, AP = Ampicillin, AMC = Amoxycillin/clavulanic acid, CRO = Ceftriaxone, CAZ = Ceftazidime, CTX = Cefotaxime, C = Chloramphenicol, TS = Co-trimoxazole, TE = Tetracycline, E = Erythromycin, CD = Clindamycin, CN = Gentamicin, CIP = Ciprofloxacin, MEM = Meropenem.

**Table 2:** Multi-drug resistant bacteria isolated from surgical site infections

**Bacteria**	**Classes of antimicrobial resisted to N(%)**						**Average N(%)**
	**R3**	**R4**	**R5**	**R6**	**R7**	**R8**	
***K. pneumoniae***	-	1(6.2)	2(12.5)	3(18.8)	8(50)	-	**14(87.5)**
***E. coli***	-	1(7.1)	1(7.1)	2(15.4)	9(62.9)	-	**13(100)**
***P. mirabilis***	-	-	1(6.3)	2(12.5)	9(56.2)	-	**12(75)**
***A. baumannii***	1(6.6)	1(6.6)	-	2(13.3)	11(73.3)	-	**15(100)**
***P. aeruginosa***	1(4)	-	-	-	-	-	**1(4)**
***P. stuartii***	-	2(28.6)	-	4(57.1)	1(14.3)	-	**7(100)**
**Other GNR**	-	-	2(12.5)	3(18.7)	6(37.5)	-	**11(36.6)**
***S. aureus***	1(5.5)	-	2(11.1)	3(16.6)	-	2(11.1)	**8(44.4)**
**CONS**	2(20)	4(40)	-	-	-	2(20)	**8(80)**
***E. faecalis***	1(20)	-	2(40)	1(20)	-	-	**4(80)**
**Total**	**6(4)**	**9(6.1)**	**9(6.1)**	**20(13.6)**	**16(10.8)**	**33(22.4)**	**93(63)**

Key: R3- R7 = resistant to 3, 4, 5, 6 or 7 classes of antimicrobials tested. Other GNR = (*M.morgannii, P.vulgaris, Enterobacter spp, Citrobacter spp*).

## Discussion

**Figure 2:**
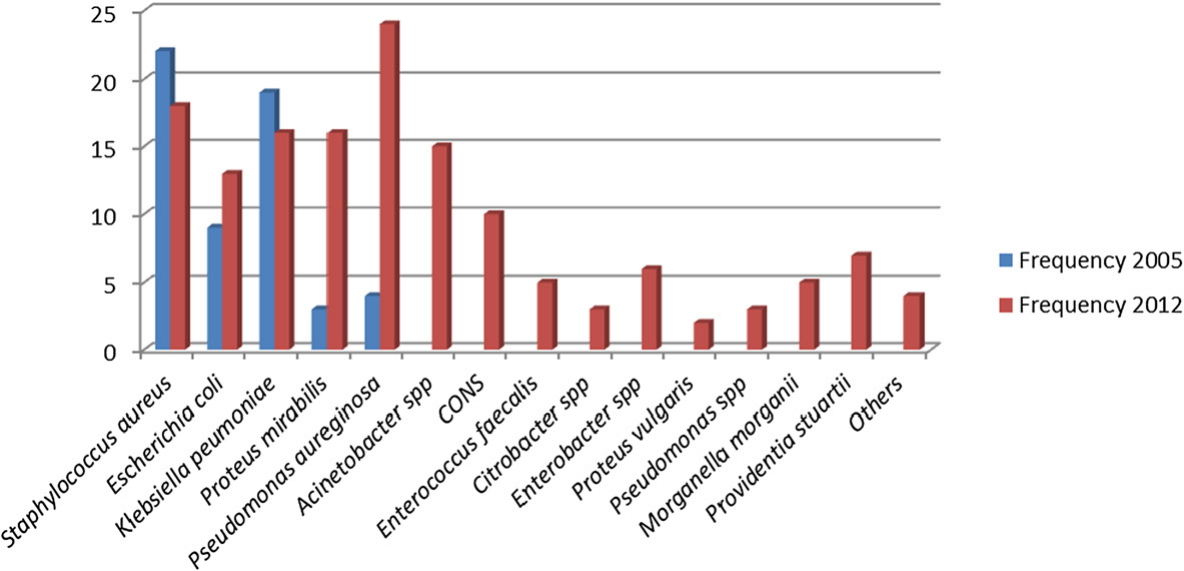
Trend of pathogenic bacterial isolated from SSI in 2005 and 2012.

This report on antimicrobial resistance from the national hospital in Tanzania demonstrates the predominance of gram negative bacterial isolates in SSIs (77.5%). Predominately *Pseudomonas aeruginosa* being the commonest followed by *K. pneumoniae* (10.8%) and *Proteus mirabilis* (10.8%). On the other hand *S.aureus* which was the commonest Gram positive organism accounted for (12.2%). This observation is in contrast to finding in previous studies from the same setting which reported Gram positive predominantly *S. aureus* as the most common SSI bacterial pathogen [7,18] (Figure 2).

With regard to antimicrobial resistance we found, among Gram negative bacteria, very high levels of resistance (73 -100%) to commonly prescribed antibiotics like ampicillin, amoxicillin/clavulanic acid, co-trimoxazole and tetracycline, which is in keeping results (32 – 100%) reported in the same hospital and in Kenya [19,20]. The level of resistance to ciprofloxacin was 20 – 56% in Enterobacteriaceae and *Acinetobacter baumannii*, showing an increase in resistance compared to (6.7 – 30%) previously reported at this hospital in 2004/2010, [11,19]. Resistance to ciprofloxacin is an early warning sign since fluoroquinolones are effective agents for treatment of gram negative bacterial infections [21].

With regard to third generation cephalosporins we found very high levels of resistance (67 -100%) among Gram negative bacteria, with most of *E coli* and *K. pneumoniae* being ESBL producing strains. These finding is in keeping with recent studies from Northern-Western Tanzania and Uganda that shows (61 – 92%) [7,22]. We noted that about 80% of patients received ceftriaxone as prophylaxis, thus favoring the emergence of resistant bacteria.

**Figure 3:**
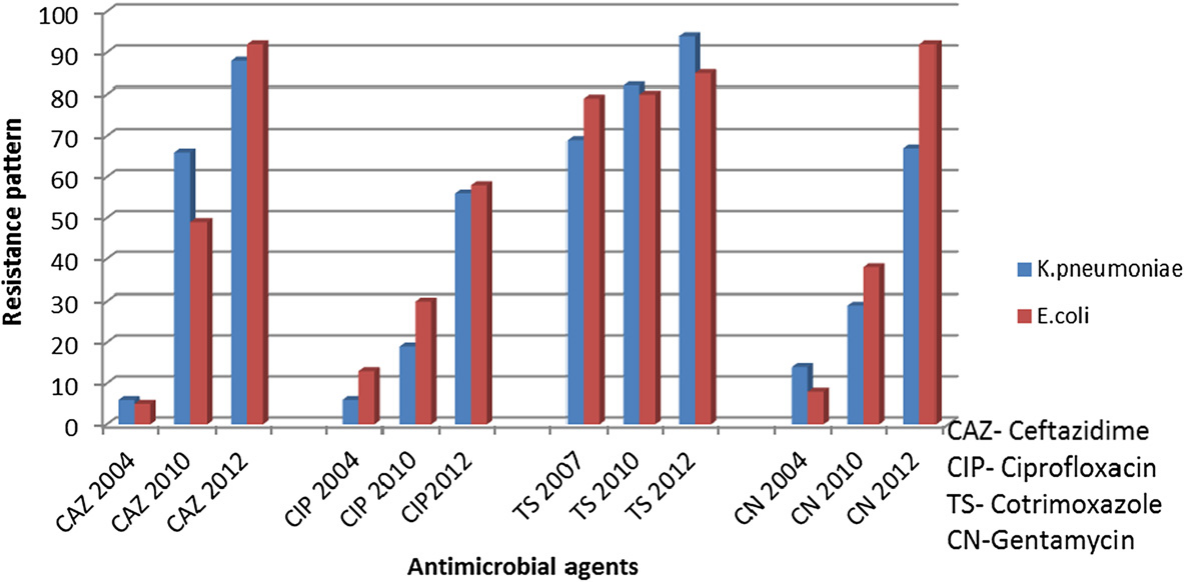
Increasing trend of resistance among Gram negative bacteria isolated from clinical isolates 2004, 2010 and 2012.

**Figure 4:**
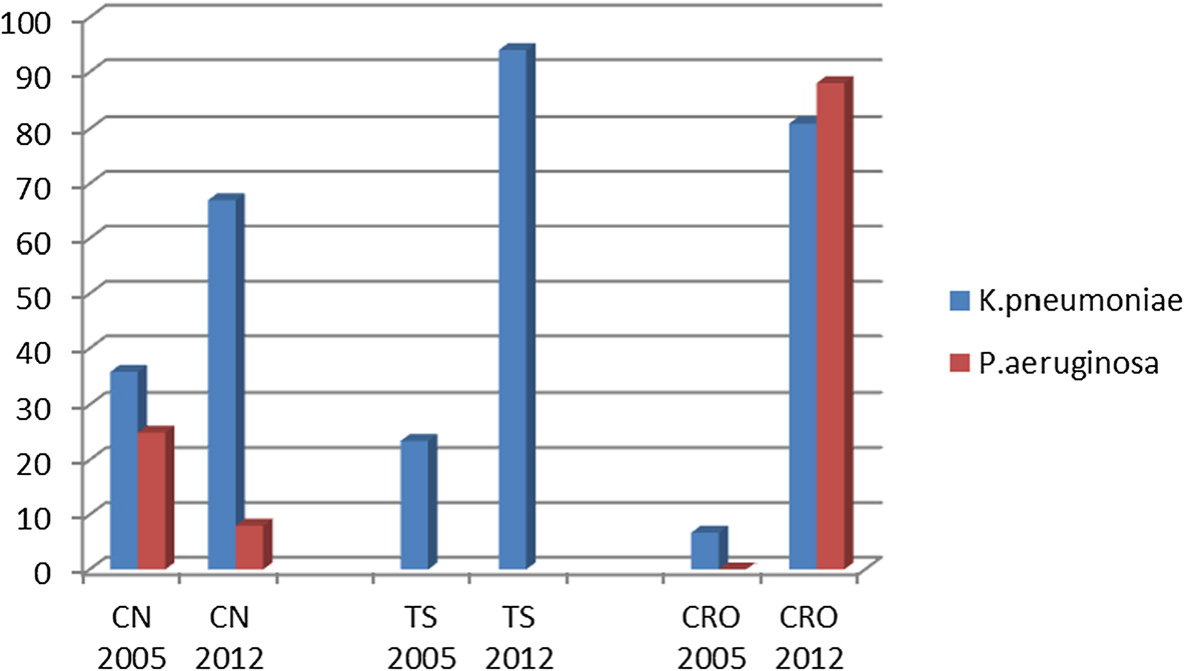
Increasing pattern of resistance to isolates from SSI at Muhimbili National Hospital 2005 and 2012.

Significantly, we note an increasing trend of resistance to cotrimoxazole, gentamicin, ceftazidime and ciprofloxacin over a period from 2004 to 2012 (Figures 3 and 4) [11,19]. In addition to the high rate of resistance to individual antibiotics, we also found significant number of MDR isolates. The overall proportion of MDR among Gram negative bacteria isolates was high (64%), this finding is in agreement with recent study from Uganda [22]. We noted that, all *Escherichia coli*, *Acinetobacter baumannii* and *Providentia stuartii* were MDR strains.

The very high levels of antibiotics resistance seen in this study could be attributed, at least in part, to inappropriate use of antimicrobial agents affects both the cost and efficacy, thus favoring the emergence of resistant bacteria.

One of the limitations of our study is cross-sectional design of the study. Also there was no patient follow up after discharge up to 30 days which is required according to CDC definition of SSI and hence some cases of SSI after discharge from hospital may be missed.

## Conclusion

The very high levels of antimicrobial resistance among bacteria causing SSIs, including resistant to more than four categories (classes), complicates the management of these infections at MNH, and could be associated with higher SSI rate and prolongation of hospital stay and increased number of drugs usage. There is a very urgent need of revising the perioperative antimicrobial prophylaxis at this national referral Hospital to provide clinicians with standardized approach to the rational, safe and effective use of antibiotics management of SSI.

## Competing interests

The authors declare that they have no competing interests.

## Authors’ contributions

JM participated in conception, design, and collection of data, analysis, interpretation and drafting of the manuscript. MIM helped to draft the manuscript. MM and SEM participated in critically revising the manuscript. SM participated in analysis and interpretation of data. EFL participated in conception, design of the study and critically revising the manuscript. All authors read and approved the final manuscript.

## Supplementary Material

Additional file 1 Demographic and clinical characteristics of patients with post-operative wound infections. Click here for file
