# Supplementary material for: Predominance of multi-drug resistant bacterial pathogens causing surgical site infections in Muhimbili national hospital, Tanzania
Source: BMC Res Notes. 2014 Aug 7;7:500. doi: 10.1186/1756-0500-7-500 (PMC4126906; doi:10.1186/1756-0500-7-500)
Supplement: Additional file 1 — Demographic and clinical characteristics of patients with post-operative wound infections. [file 1756-0500-7-500-S1.docx]

**Additional file 1**

**Table 1: Demographic and clinical characteristics of patients with post-operative wound infections**

| **Variables** | **No (%)** |
| --- | --- |
| **Age (years)** | |
| Mean (SD) | 36.5 (16.6) |
| Median | 32 |
| Range | 13-81 |
| **Sex** | |
| Male | 53(53) |
| Female | 47(47) |
| **Surgical department** | |
| General surgery | 29 (29) |
| Obstetrics/Gynecology | 25 (25) |
| Muhimbili Orthopedic Institute | 46 (46) |
| **Type of surgery** |  |
| Emergency | 80 (80) |
| Elective | 20 (20) |
| **Type of incision** | |
| Clean | 30 (30) |
| Clean contaminated | 5 (5) |
| Contaminated | 37 (37) |
| Dirty | 28 (28) |
| **Type of operation** | |
| Surgical Debridement + External Fixation | 27 (27) |
| Caesarian section | 15 (15) |
| Laparotomy | 25 (25) |
| Open reduction + Internal fixation | 9 (9) |
| Amputation | 8 (8) |
| Surgical debridement | 6 (6) |
| Others | 10 (10) |
| **Previous antibiotics exposure within 1 month** | |
| Yes | 95(95) |
| No | 5(5) |
| **Previous hospitalization within 6 months** | |
| Yes | 75 (75) |
| No | 25 (25) |
